# Controlled Preparation of Nanoparticle Gradient Materials by Diffusion

**DOI:** 10.3390/nano9070988

**Published:** 2019-07-09

**Authors:** Andreas Spinnrock, Max Martens, Florian Enders, Klaus Boldt, Helmut Cölfen

**Affiliations:** 1 Department of Chemistry, University of Konstanz, 78457 Konstanz, Germany; 2 Department of Applied Science (Materials Science & Engineering), Fontys University of Applied Science, 5612 MA Eindhoven, The Netherlands

**Keywords:** composites, diffusion, functional materials, gradients, nanoparticles

## Abstract

Nanoparticle gradient materials combine a concentration gradient of nanoparticles with a macroscopic matrix. This way, specific properties of nanoscale matter can be transferred to bulk materials. These materials have great potential for applications in optics, electronics, and sensors. However, it is challenging to monitor the formation of such gradient materials and prepare them in a controlled manner. In this study, we present a novel universal approach for the preparation of this material class using diffusion in an analytical ultracentrifuge. The nanoparticles diffuse into a molten thermoreversible polymer gel and the process is observed in real-time by measuring the particle concentrations along the length of the material to establish a systematic understanding of the gradient generation process. We extract the apparent diffusion coefficients using Fick’s second law of diffusion and simulate the diffusion behavior of the particles. When the desired concentration gradient is achieved the polymer solution is cooled down to fix the concentration gradient in the formed gel phase and obtain a nanoparticle gradient material with the desired property gradient. Gradients of semiconductor nanoparticles with different sizes, fluorescent silica particles, and spherical superparamagnetic iron oxide nanoparticles are presented. This method can be used to produce tailored nanoparticle gradient materials with a broad range of physical properties in a simple and predictable way.

## 1. Introduction

Nanoparticles are of great scientific and commercial interest because they often possess unique size-dependent physical and chemical properties. Many of them can nowadays be produced at large scales [1,2]. It is desirable to transfer their properties to macroscopic materials. In polymer nanocomposites, the nanoparticles are embedded in a surrounding macroscopic polymer matrix [3,4,5]. Examples are polymer silver composites [6,7], polymer gold composites [8,9], polymer copper composites [10,11], and polymer semiconductor composites [12,13]. Nanoparticle gradient materials are a unique class of functional nanoparticle composite materials. They obtain a concentration gradient of the nanoparticles that leads to a spatial physical property gradient (e.g., optical, electrical, mechanical or magnetic) in the material. Thus, they have the potential for applications in optics (e.g., gradient lenses for microscopes and cameras), electronics (e.g., for micro- and nano-electromechanical systems (MEMS and NEMS)), magnetic devices (e.g., magnetic switches) and sensors. Previously, nanoparticle property gradients have been generated in functionally graded nanomaterials by thickness-gradients [14]. For example, functionally graded nanobeams for small device applications in MEMS and NEMS have been produced and their thermoelastic behavior has been modeled by stress-driven nonlocal integral modelling [15,16]. Preparation methods for polymeric gradient materials are photopolymerization [17], selective laser sintering [18,19], corona discharge [20], layer-by-layer assembly [21], 3D printing [22], centrifugal casting [23], and sedimentation in analytical ultracentrifuges [24]. Disadvantages of the established techniques are either that the gradients cannot be simulated in advance and detected in real-time during fabrication or that their upscale ability is limited due to tedious and complicated fabrication setups. 

A good understanding of the formation process is necessary to overcome these drawbacks. Gradient materials that are prepared by diffusion of nanoparticles are good candidates for such investigations because the diffusion of molecules and nanoparticles is well established and has been described mathematically by Fick more than 150 years ago [25]. Ion diffusion has been used to generate gold nanoparticle gradient structures [26,27]. Diffusion behavior of nanoparticles in polymers was also investigated and it was shown that the diffusion coefficient is dependent on the radius of gyration of the polymer and cannot always be described by the Stokes–Einstein relation [28,29,30]. To our knowledge, diffusion of nanoparticles has not been used before to produce nanoparticle gradients materials in a systematic way.

In this study, we generate materials with a concentration gradient in an analytical ultracentrifuge by diffusion of nanoparticles into a thermoreversible gel matrix. By monitoring the nanoparticle diffusion and gradient formation in real-time with the optics of the analytical ultracentrifuge, we are able to extract the apparent diffusion coefficients. With this approach, we can simulate the diffusion behavior of the nanoparticles and gain control over the resulting material properties. That way, tailored nanoparticle gradient materials can be produced.

## 2. Materials and Methods 

### 2.1. General Procedure

**Figure 1:**
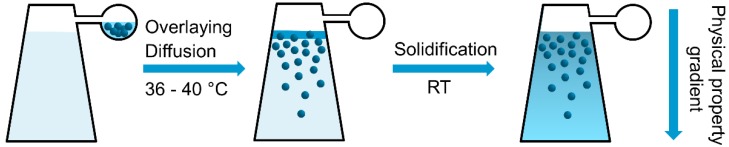
Schematic view of the preparation of a nanoparticle gradient material in the sample sector of an analytical ultracentrifugation band-forming cell. The nanoparticles (blue spheres) are overlaid at low rotational speed and diffuse into the molten gelatin gel. After achieving the desired nanoparticle concentration gradient, the cell is cooled to room temperature to solidify the gelatin and fix the gradient in the gel phase. A physical property gradient inside the material is obtained.

The materials are prepared by overlaying experiments using band-forming cells in an analytical ultracentrifuge. Dispersed nanoparticles were filled into the reservoir of a band-forming centerpiece (Figure S1). A thermoreversible material (gelatin) was filled into the sample sector and the reference sector of the centerpiece. The cells were heated in the centrifuge to melt the material (36–40 °C). Upon speeding up the rotor, the nanoparticles were overlaid onto the sample sector through thin capillaries (Figure 1) [31,32]. The nanoparticles started to diffuse into the liquid gelatin to form a concentration gradient. No sedimentation was taking place when the sedimentation coefficients of the particles were small and the rotational speed was low. The concentration gradient was continuously detected in real-time with the integrated optics of the centrifuge. When the targeted gradient was achieved, the liquid gelatin was cooled down to room temperature to solidify and the desired nanoparticle gradient material was obtained. The advantages of the usage of the analytical ultracentrifuge were that the gradient generation can be detected and that the overlaying and thus the diffusion process was started at a desired, defined point in time.

### 2.2. Materials

CdO (>99.5%), tri-n-octylphosphine (TOP, 97%), tri-n-octylphosphine oxide (TOPO, 99%), thioglycolic acid (TGA, 98%), gelatin (Type B, ~225 g Bloom), m-Cresol, deuterium oxide, and methanol were purchased from Sigma-Aldrich. N-Octadecylphosphonic acid (ODPA, >99%) was purchased from PCI Synthesis, selenium shot (99.999%) from Alfa Aesar, potassium hydroxide (85–100%) from VWR and toluene (>99.5%) from Carl Roth. All chemicals were used as received without further purification.

### 2.3. Synthesis of Stock Gelatin Gel

The stock gelatin gel was prepared by adding gelatin (14 g) to milliQ water (86 g). The suspension was swollen for 24 h at RT and m-Cresol solution (2.1 mL, 5 wt% in Methanol) was added. The mixture was heated to 50 °C for 2 h under continuous stirring. After heating, the gel was stored in the fridge. The stock gelatin gel was produced by adding deuterium oxide (250 µL) to the gel (1 g).

### 2.4. Nanoparticle Synthesis

CdSe cores of various sizes were prepared by injecting tri-n-octylphosphine selenide into a solution of cadmium phosphonate in tri-n-octylphosphine oxide at 370 °C following the procedure reported by Carbone and co-workers [33].

CdSe quantum dots were transferred into water by employing the protocol for ligand exchange against thioglycolic acid described by Sánchez–Paradinas et al. [34]. In short, thioglycolic acid was added to a 0.1 M KOH solution in methanol and the mixture was added to a dilute solution of CdSe quantum dots in hexane. The two phases were vigorously shaken and then centrifuged. The supernatant was discharged and excess KOH was removed by washing with MeOH before redispersing the dots in water.

The Rhodamine B isothiocyanate-incorporated silica nanoparticles were prepared as described previously [35]. Briefly, a fluorescent core was produced by crosslinking 3-aminopropyltriethoxysilane with rhodamine B isothiocyanate. Hydrolyzed tetraethylorthosilicate was crosslinked to form a shell around the fluorescent cores. Then polyethylene glycol-silane (PEG-silane) was linked to the surface of the nanoparticles as a steric stabilizer.

The superparamagnetic iron oxide nanoparticles were prepared by thermal decomposition of an iron oleate complex in the presence of oleic acid as described previously [36]. 

### 2.5. Preparation of Nanoparticle Gradient Materials

Aqueous nanoparticle dispersion (10 µL) was filled in the reservoir of the centerpiece and the centrifugation cell was assembled. Stock gelatin gel (1 g) was stored in a drying oven for 30 min at 50 °C. 240 µL of the liquid stock gelatin gel was filled into the sample sector and 300 µL of the liquid stock gelatin gel was filled into the reference sector. The centrifuge was set to a speed of 3000 rpm (726 RCF(max)) and a temperature of 36 °C or 40 °C depending on the nanoparticles. When the desired absorbance/concentration gradient was detected, the target temperature was set to 15 °C and the samples were cooled for 2 h to obtain the nanoparticle gradient material.

### 2.6. Instrumentation

Analytical Ultracentrifugation was carried out on a Beckman–Coulter XL-A/XL-I. The samples were run in 12 mm charcoal filled Epon Beckman Band forming centerpieces of the Vinograd type. Preparative ultracentrifugation was carried out on a Beckman Optima L-70 ultracentrifuge with a SW 55 Ti Swinging-Bucket Rotor in 3.5 mL Thickwall Polyallomer tubes. The temperature was equilibrated for 3 h to minimize temperature gradients in the centrifugation tube. UV/Vis absorbance spectra were acquired using an Agilent Cary 60 spectrometer and an Ocean Optics USB-DT light source with an Ocean Optics USB2000+ spectrometer. Transmission electron microscopy (TEM) images were taken on a Zeiss TEM Libra 120 operating at 120 kV. High-resolution TEM micrographs were obtained using a Jeol JEM 2200FS transmission electron microscope operated at 200 kV. Samples were prepared by drop casting 10 µL of dilute sample solution in toluene onto a carbon-coated copper grid (Quantifoil). All calculations and simulations were performed with MATLAB.

## 3. Results and Discussion

### 3.1. Nanoparticle Gradient Materials with Semiconductor Nanoparticles

**Figure 2:**
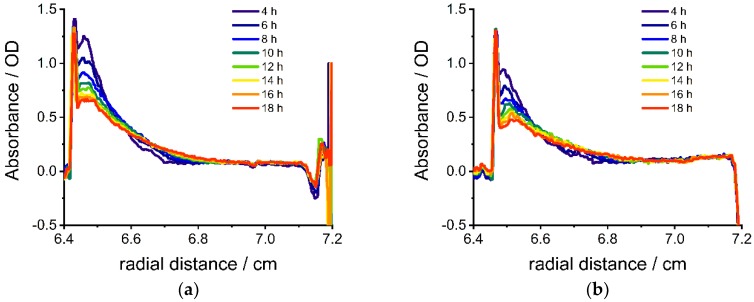

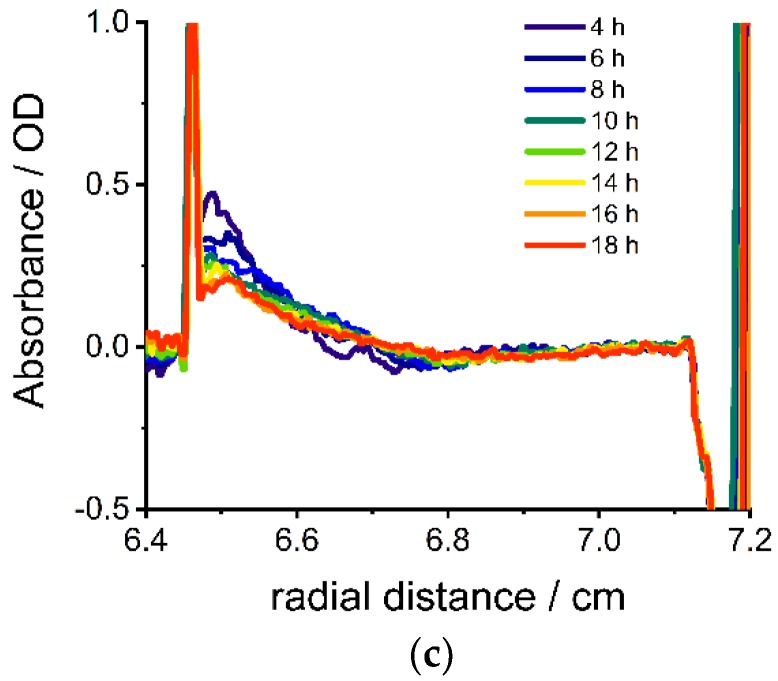
Absorbance profile of gelatin with small (**a**) (d = 2.8 nm, λ = 482 nm), medium-sized (**b**) (d = 3.2 nm, λ = 500 nm) and large (**c**) (d = 3.8 nm, λ = 500 nm) CdSe nanoparticles against radial distance from axis of rotation at different times after overlaying at 36 °C.

The diffusion of nanoparticles with specific optical properties (absorbance or refractive index) into the gelatin can be monitored in an analytical ultracentrifuge. CdSe nanoparticles show specific size-dependent optical and electronic properties due to quantum-size-confinement [37]. They are promising for gradient materials with optical (color) and electronic property gradients. CdSe nanoparticles with three different sizes were investigated: small, yellow–orange nanoparticles with a diameter of 2.8 nm, medium-sized, orange nanoparticles with a diameter of 3.2 nm and large, red nanoparticles with a diameter of 3.8 nm (Photograph Figure S2, UV/Vis absorbance spectra Figure S3, transmission electron microscopy (TEM) images Figures S4–S6). CdSe nanoparticles of such small sizes do not sediment in the centrifuge at low rotational speeds (3000 rpm, 726 RCF(max)), because of their small sedimentation coefficient. A reference sedimentation velocity experiment with the large CdSe nanoparticles (d = 3.8 nm) at 40,000 rpm (129,000 RCF(max)) is shown in Figure S7. Nearly no sedimentation of the particles was detected, even though the centrifugal force is more than 175 times the centrifugal force at 3000 rpm. Therefore, only diffusion contributes to the change of concentration during the experiment. Figure 2 shows absorbance profiles during the diffusion of the CdSe nanoparticles with different sizes into gelatin after different diffusion times. A typical broadening of the concentration by diffusion over time is detected. Smaller nanoparticles (Figure 2a) diffuse faster than bigger nanoparticles (Figure 2c). The sharp peak at low radial distances from the axis of rotation (between 6.4 and 6.45 cm) corresponds to the meniscus of the air/sample interface. No scans during the first 3.5 h are shown, because of the black band at the gelatin-nanoparticle dispersion interface (Figure S8). This phenomenon is caused by a steep refractive index gradient [38] between water and gelatin and disappears when the overlaying water has diffused into the gelatin. The small shift in the absorbance baseline for the medium-sized nanoparticles (OD = 0.1) can be explained by polydisperse aggregates that are formed during storage of the nanoparticles in aqueous solution. To avoid this shift, freshly prepared CdSe nanoparticles have to be used. However, purposeful aggregation can be utilized for a baseline shift if such concentration gradients are desired. The formation of the concentration gradient can be detected for all three nanoparticle samples in real-time.

The apparent diffusion coefficients of the particles in the molten gelatin are calculated to allow prediction of the nanoparticle gradient formation. The changes of the concentration by diffusion over time are described by Fick’s second law of diffusion [25]

(1) $$\frac{\partial c\left(x,t\right)}{\partial t}=D\frac{\partial^{2}c\left(x,t\right)}{\partial x^{2}},$$

in which the accumulation is proportional to the diffusion coefficient D and the second derivative of the concentration. When the particles do not reach the bottom of the cell and thus no back-diffusion is taking place, Fick’s second law can be solved for a finite source in a semi-infinite medium [39].

(2) $$A\left(x,t\right)=\frac{A_{0}}{2}\left(\mathrm{erf}(\frac{h-x}{\sqrt{4Dt}})+\mathrm{erf}\left(\frac{h+x}{\sqrt{4Dt}}\right)\right).$$

Here A(x,t) is the absorbance at distance x and time t, $A_{0}$ is the absorbance of the overlaying solution and h is half of the thickness of the overlaying solution. The absorbance is taken from UV/Vis absorbance measurements and the thickness is calculated from the volume of the overlaying solution divided by the length and width of the cell sector. Using Equation (2), the absorbance profiles from Figure 2 are fitted to get the apparent diffusion coefficients of the nanoparticles.

**Figure 3:**
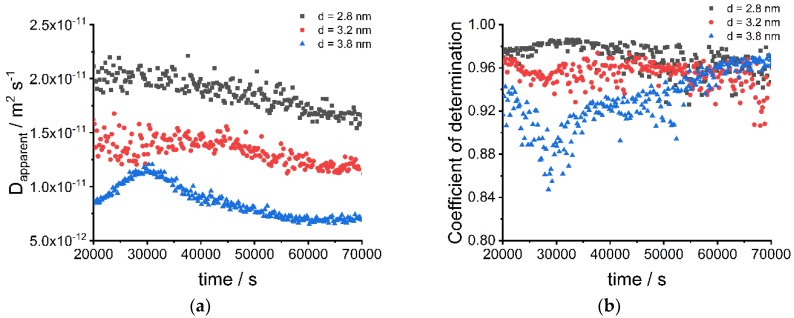
(**a**) Apparent diffusion coefficients of small (d = 2.8 nm), medium-sized (d = 3.2 nm) and large (d = 3.8 nm) CdSe nanoparticles fitted from diffusion experiments at different times; (**b**) Coefficient of determination from fitting functions of diffusion experiments of small (d = 2.8 nm), medium-sized (d = 3.2 nm) and large (d = 3.8 nm) CdSe nanoparticles.

Figure 3 shows the apparent diffusion coefficients (a) and the coefficients of determination (b) for the fits at different times and different sizes of CdSe nanoparticles. As expected, larger nanoparticles show smaller apparent diffusion coefficients and vice versa. The increase of the apparent diffusion coefficient around 30,000 s for the large nanoparticles most likely results from a fitting problem because the coefficients of determination are lower at this time interval. The investigated nanoparticle-system is a complex system with interactions between nanoparticles, ligands, and gelatin. Gelatin can act as a ligand for the nanoparticles. These effects are not directly considered in the simulation model. Thus, the diffusion coefficient is only an apparent diffusion coefficient and cannot be described by the Stokes–Einstein relation. Nevertheless, the model is universally applicable for different nanoparticle systems. Moreover, water diffuses in the gelatin material and leads to a gradient of viscosity until the water is distributed homogenously in the gelatin. However, the effect on the apparent diffusion coefficients is small and thus does not need to be considered for the simplified simulation model. The apparent averaged diffusion coefficients for the different sizes of nanoparticles are 1.90 × 10^−11^ m^2^·s^−1^ (small nanoparticles) 1.33 × 10^−11^ m^2^·s^−1^ (medium-sized nanoparticles) and 8.6 × 10^−12^ m^2^·s^−1^ (large nanoparticles).

**Figure 4:**
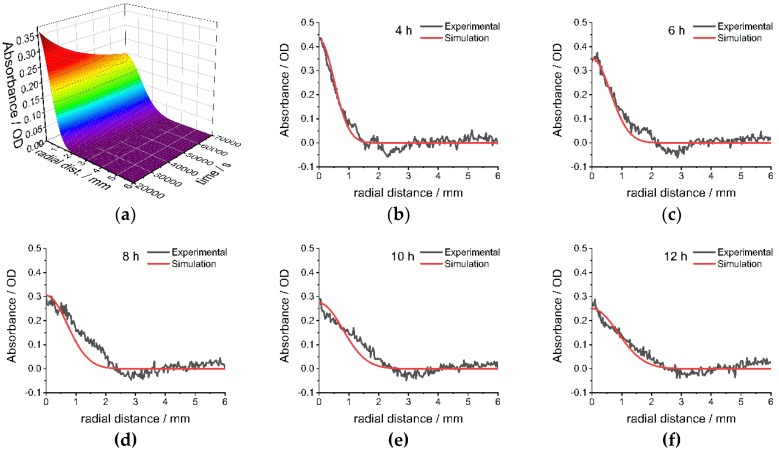

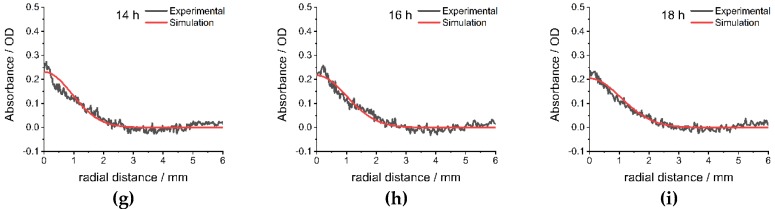
(**a**) Simulated absorbance profile after overlaying of large CdSe nanoparticles (d = 3.8 nm) over time; Comparison of simulated and experimental absorbance profiles after overlaying of large CdSe nanoparticles (d = 3.8 nm) after (**b**) 4 h, (**c**) 6 h, (**d**) 8 h, (**e**) 10 h, (**f**) 12 h, (**g**) 14 h, (**h**) 16 h and (**i**) 18 h at λ = 500 nm at 36 °C. Radial distance is distance from the top of the polymer melt.

The extracted apparent diffusion coefficients are applied to simulate the gradient formation at different times in other experiments using Equation (2). Figure 4a shows the simulated diffusion process of the large CdSe nanoparticles at 36 °C and by that flattening of the absorbance gradient (λ = 500 nm). Comparison of simulated and experimental data after different diffusion times shows good agreement (Figure 4b–i, residuals between simulation and experiments Figure S9). In the time range between 6 h and 10 h, the model overestimates the absorbance at distances < 1 mm and underestimates the absorbance at distances > 1 mm. Small deviations between experimental and simulated data can be explained by the complexity of the interactions between gelatin, surfactants, and nanoparticles, which are only considered indirectly by using the apparent diffusion coefficient. Nevertheless, the model can be used to predict the generation of nanoparticle gradients in diffusion experiments with good accuracy, especially after long diffusion times (>10 h). Comparisons of the absorbance profile after simulated and experimental diffusion for the small CdSe nanoparticles (Figure S10) and medium-sized CdSe nanoparticles (Figure S11) also show good agreement with minor deviations at small distances from the overlaying interface.

### 3.2. Nanoparticle Gradient Materials with Fluorescent Silica Nanoparticles

**Figure 5:**
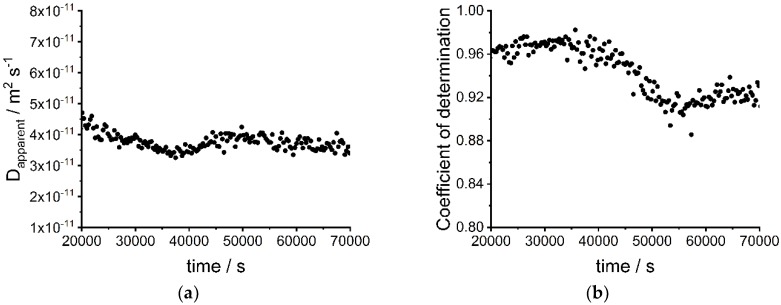
(**a**) Apparent diffusion coefficients of spherical Rhodamine B isothiocyanate-incorporated silica nanoparticles (RITC-SiNPs) fitted from diffusion experiments at different times; (**b**) coefficient of determination from fitting functions of diffusion experiments of RITC-SiNPs.

**Figure 6:**
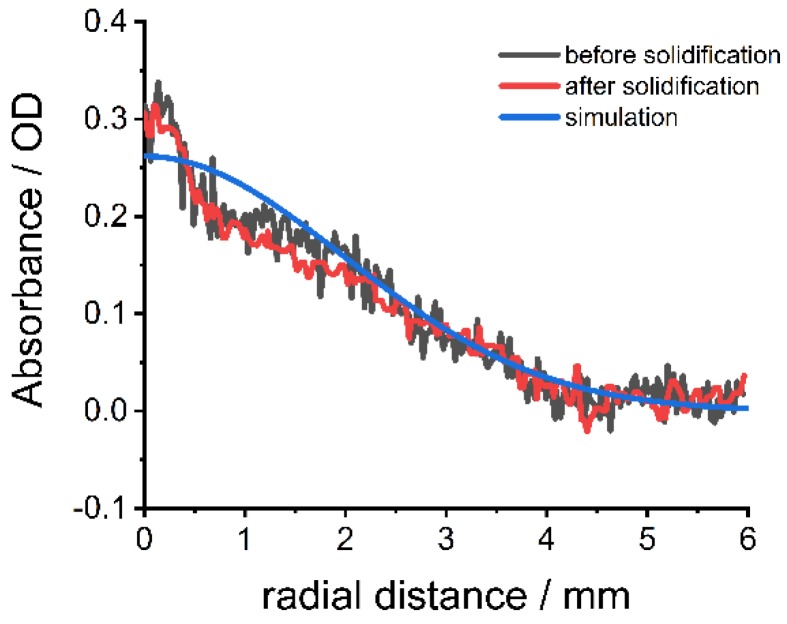
Simulated absorbance profile and absorbance profiles before and after solidification of gelatin nanoparticle gradient material with RITC-SiNPs at λ = 550 nm. Radial distance is the distance from the top of the melt/material.

**Figure 7:**
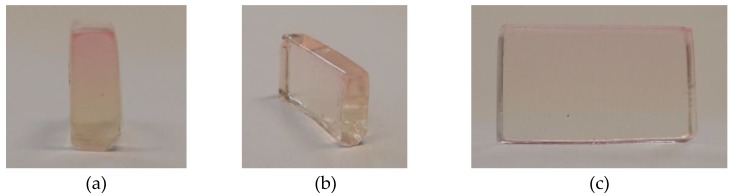
Photographs of gelatin nanoparticle gradient material with a gradient of RITC-SiNPs from three different views: frontal view in (**a**), oblique view in (**b**) and side view in (**c**). The dimensions of the material are 1.2 cm × 0.7 cm × 0.3 cm.

The presented system is not limited to quantum dot nanoparticles but can be applied to different nanoparticles, if they are dispersible in water. Fluorescence dye-labeled silica nanoparticles have tunable optical (absorbance and fluorescence) properties depending on the dye. Moreover, the size of the nanoparticles can be adjusted over a broad range without losing the optical properties [40]. Spherical Rhodamine B isothiocyanate-incorporated silica nanoparticles (RITC-SiNPs) with an absorbance maximum at 550 nm (Figure S12) and a diameter of 25 nm (TEM image Figure S13) were used for the preparation of nanoparticle gradient materials. Because of their larger size and thus lower diffusion coefficient, the diffusion experiments were performed at a higher temperature (40 °C) to lower the viscosity of the gelatin melt. The absorbance of the nanoparticles at different times after overlaying was detected in the analytical centrifuge at λ = 550 nm and 3000 rpm (726 RCF(max)) (Figure S14) and shows the expected broadening by diffusion and no contribution of sedimentation. Sedimentation would lead to a shift of the absorbance maximum. The apparent diffusion coefficient was fitted by Equation (2) (Figure 5a) with good coefficients of determination (Figure 5b). Despite the bigger size, the averaged apparent diffusion coefficient D = 3.8 × 10^−11^ m^2^·s^−1^ was higher than the apparent diffusion coefficients of the CdSe nanoparticles (Figure 3a) because of the higher temperature (40 °C instead of 36 °C) and thus lower viscosity of the gelatin. The apparent diffusion coefficient was used for the simulation of the change in absorbance over time (Figure S15). A material with a defined nanoparticle gradient was produced by stopping the centrifuge and cooling down to room temperature after 14.4 h. The concentration gradient was retained during the solidification process and a material with the desired simulated absorbance gradient was produced (Figure 6, residuals: Figure S16). The dimensions of the material are 1.2 cm × 0.7 cm × 0.3 cm. An optical color gradient that is caused by the nanoparticle concentration gradient is visible by the naked eye (Figure 7). 

Gelatin was used as the test system here, but other thermoreversible polymers can also be potentially used as the polymer matrix. For each nanoparticle/matrix system the apparent diffusion coefficient has to be determined once to enable further simulations. Then, nanoparticle gradient materials with different gradients can be prepared. Thus, nanoparticle gradient materials with gradients of different nanoparticles and different polymer matrices can be produced in a predictable and detectable way.

### 3.3. Nanoparticle Gradient Materials by a Combination of Diffusion and Sedimentation

**Figure 8:**
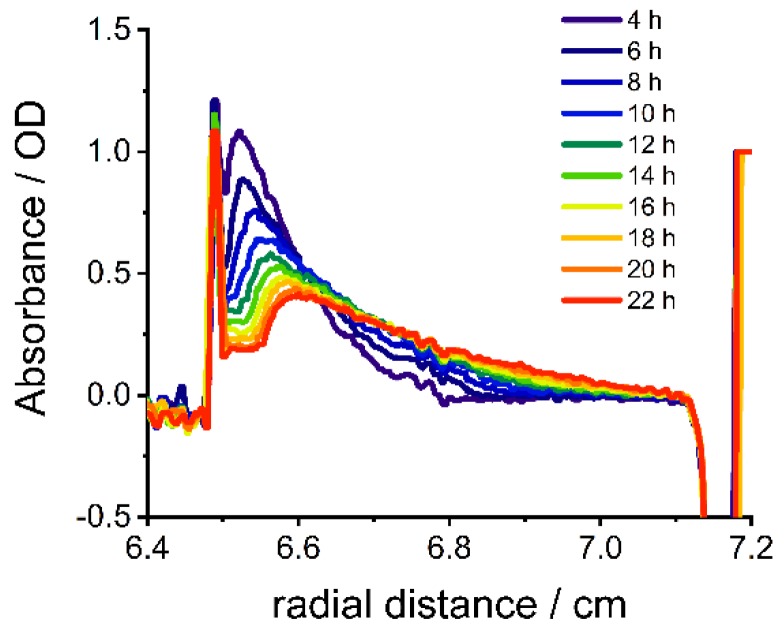
Absorbance profile of gelatin with superparamagnetic iron oxide nanoparticles (SPIONs) against radial distance from axis of rotation at different times after overlaying at λ = 450 nm at 40 °C.

A larger variety of concentration profiles is accessible when gradient formation by diffusion is combined with sedimentation. A contribution of sedimentation to the gradient formation was caused by a higher gravitational force acting on the particles. Gravitational force can be increased either by increasing the rotational speed or by using particles with a higher sedimentation coefficient. Previously, the controlled fabrication of nanoparticle gradients by sedimentation was described [24]. Superparamagnetic iron oxide nanoparticles (SPIONs) are interesting for gradient materials with specific magnetic properties for example in magnetic switches. In Figure 8 a band forming experiment and subsequent diffusion of SPIONs (TEM image Figure S17) in gelatin at 3000 rpm (726 RCF(max)) is presented. The absorbance maximum shifts from a radial distance from the axis of rotation of 6.5 cm to 6.6 cm over time, due to sedimentation of the particles like in a classical band sedimentation experiment [32]. Thus, a wider variety of gradients is accessible, when diffusion and sedimentation are combined. The sedimentation coefficient of the particles can be extracted (24.8 S for SPIONs) from the shift of the absorbance maximum [41].

(3) $$\ln\left(\frac{r_{bnd}}{r_{m}}\right)=s\omega^{2}t.$$

Here $r_{bnd}$ is the radial position of the maximum, $r_{m}$ is the radial position of the meniscus, s is the sedimentation coefficient, ω is the angular velocity and t is the experimental time. Using Equation (3) the shift of the maximum at different rotational speeds and times can then be simulated. However, the change in concentration cannot be described by a solution of Fick’s second law for a finite source in a semi-infinite medium (Equation (2)) anymore, because sedimentation also contributes to the change of the concentration. Numerical approximations of the Lamm equation [42] can potentially be used to describe the generated gradients, when the sedimentation and diffusion coefficients are known.

### 3.4. Upscaling

**Figure 9:**
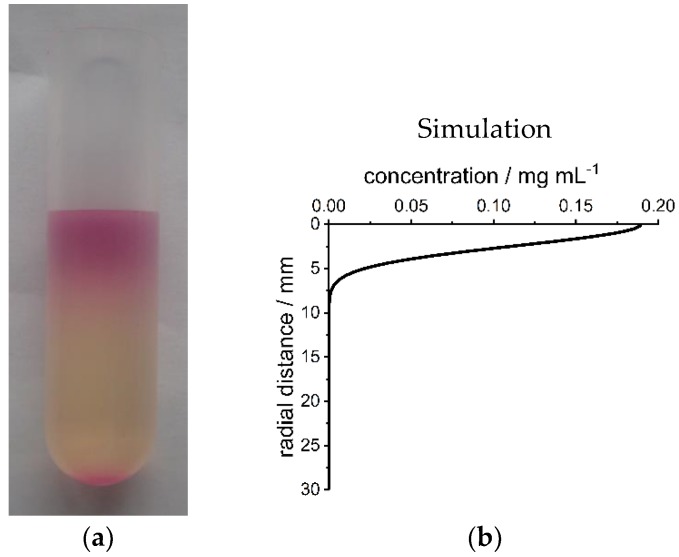
(**a**) Photograph of gelatin nanoparticle gradient material with a gradient of RITC-SiNPs obtained after centrifugation in a preparative ultracentrifuge at 3000 rpm (1094 RCF(max)) and 40 °C for 20 h. The size of the material is 3 cm length and 1 cm diameter; (**b**) simulated concentration gradient of the material from (**a**). The scales of (**a**) and (**b**) are identical. Radial distance is distance from the top of the material.

Upscaling of the controlled fabrication process from analytical centrifuges to preparative centrifuges is very simple when the apparent diffusion coefficient of the particles is known. We show this for a gradient material of RITC-SiNPs in gelatin (Figure 9) with a length of 3 cm and a diameter of 1 cm. The light pink color at the bottom of the tube is caused by aggregated nanoparticles that sedimented. Nanoparticle gradient materials with defined properties can be produced in any centrifuge in any lab. In principle, diffusion takes place in every reaction vessel and thus no centrifuge is necessary. However, convection that can be caused by temperature and density gradients can lead to changes in the concentration gradient. This is prevented by centrifugation. Thus, nanoparticle gradient materials with various property gradients can be produced on a large scale (mg up to multi-g scale and mm up to m gradient thickness, depending on the size of the reaction vessel).

## 4. Conclusions

In conclusion, a novel method for the predictable generation of gradient polymer nanocomposites has been established. A nanoparticle dispersion is overlaid on a polymer melt and controlled diffusion takes place. The formation of the concentration gradient is detected in real-time by using the optics of an analytical ultracentrifuge. That way, a systematic understanding and simulations of the diffusion process and gradient formation are established. After cooling down, the polymer melt solidifies and the desired gradient polymer nanocomposite material is obtained. Different nanoparticles lead to gradients of different physical properties (e.g., absorbance gradients with semiconductor and dye-labeled silica nanoparticles or conductivity gradients with metal nanoparticles). Such materials are promising for applications in optics, electronics, and sensors. The diffusion process can take place in any centrifuge. Thus, the method can potentially be used in any lab in the world to produce tailored polymer nanoparticle gradient materials.

## Supplementary Materials

The following are available online at https://www.mdpi.com/2079-4991/9/7/988/s1, Figure S1: Photograph of a band-forming centerpiece with reference sector (left), sample sector (right) and reservoir, Figure S2: Photograph of CdSe nanoparticle dispersions. From left to right: small nanoparticles (d = 2.8 nm), medium-sized nanoparticles (d = 3.2 nm) and large nanoparticles (d = 3.8 nm), Figure S3: UV/Vis absorbance spectra of spherical CdSe nanoparticles with different diameters in toluene, Figure S4: HR-TEM-Images of small spherical CdSe nanoparticles with a diameter of 2.8 nm, Figure S5: HR-TEM-Images of medium-sized spherical CdSe nanoparticles with a diameter of 3.2 nm, Figure S6: HR-TEM-Images of large spherical CdSe nanoparticles with a diameter of 3.8 nm, Figure S7: Absorbance profile of gelatin with large (d = 3.8 nm, λ = 546 nm) CdSe nanoparticles against radial distance from axis of rotation at different times in a sedimentation velocity experiment at 40,000 rpm (129,000 RCF(max)), Figure S8: Absorbance profiles of gelatin with small (a) (d = 2.8 nm, λ = 482 nm), medium-sized (b) (d = 3.2 nm, λ = 500 nm) and large (c) (d = 3.8 nm, λ = 500 nm) CdSe nanoparticles against radial distance from axis of rotation at different times after overlaying at early times at 36 °C. Black band phenomena are observed, Figure S9: Absorbance residuals between simulation and experimental detection for gelatin with large (d = 3.8 nm) CdSe nanoparticles after (a) 4 h, (b) 6 h, (c) 8 h, (d) 10 h, (e) 12 h, (f) 14 h, (g) 16 h and (h) 18 h at λ = 500 nm at 36 °C. Radial distance is distance from the top of the polymer melt, Figure S10: (a) Simulated absorbance profile after overlaying of small CdSe nanoparticles (d = 2.8 nm) over time; Comparison of simulated and experimental absorbance profiles after overlaying of small CdSe nanoparticles (d = 2.8 nm) after (b) 4 h, (c) 6 h, (d) 8 h, (e) 10 h, (f) 12 h, (g) 14 h, (h) 16 h and (i) 18 h at λ = 482 nm at 36 °C. Absorbance residuals between simulation and experimental detection for gelatin with small (d = 2.8 nm) CdSe nanoparticles after (j) 4 h, (k) 6 h, (l) 8 h, (m) 10 h, (n) 12 h, (o) 14 h, (p) 16 h and (q) 18 h at λ = 482 nm at 36 °C, Figure S11: (a) Simulated absorbance profile after overlaying of medium-sized CdSe nanoparticles (d = 3.2 nm) over time; Comparison of simulated and experimental absorbance profiles after overlaying of medium-sized CdSe nanoparticles (d = 3.2 nm) after (b) 4 h, (c) 6 h, (d) 8 h, (e) 10 h, (f) 12 h, (g) 14 h, (h) 16 h and (i) 18 h at λ = 500 nm at 36 °C. Absorbance residuals between simulation and experimental detection for gelatin with small (d = 2.8 nm) CdSe nanoparticles after (j) 4 h, (k) 6 h, (l) 8 h, (m) 10 h, (n) 12 h, (o) 14 h, (p) 16 h and (q) 18 h at λ = 500 nm at 36 °C. Radial distance is distance from the top of the polymer melt, Figure S12: UV/Vis absorbance spectra of RITC-SiNPs (d = 25 nm) in water, Figure S13: TEM-images of spherical Rhodamine B isothiocyanate-incorporated silica nanoparticles (RITC-SiNPs) with a diameter of 25 nm, Figure S14: Absorbance profile of gelatin with RITC-SiNPs against radial distance from axis of rotation at different times after overlaying at λ = 550 nm at 40 °C, Figure S15: Simulated absorbance profile after overlaying of small RITC-SiNPs (d = 25 nm) over time. Radial distance is distance from the top of the polymer melt, Figure S16: Absorbance residuals between simulation and experimental detection for nanoparticle gradient material with RITC-SiNPs (d = 25 nm) before and after solidification at λ = 550 nm. Radial distance is distance from the top of the polymer melt, Figure S17: TEM-Images of spherical superparamagnetic iron oxide nanoparticles (SPIONs) with a diameter of 19 nm.
