# Supplementary material for: Controlled Preparation of Nanoparticle Gradient Materials by Diffusion
Source: Nanomaterials (Basel). 2019 Jul 9;9(7):988. doi: 10.3390/nano9070988 (PMC6669869; doi:10.3390/nano9070988)
Supplement: Supplementary file 1 [file nanomaterials-09-00988-s001.pdf]

nanomaterials

Supplementary Materials

Controlled Preparation of Nanoparticle Gradient Materials by Diffusion

Andreas Spinnrock [1], Max Martens [2], Florian Enders [1], Klaus Boldt [1] and Helmut Cölfen [1,*]

Supplementary Figures

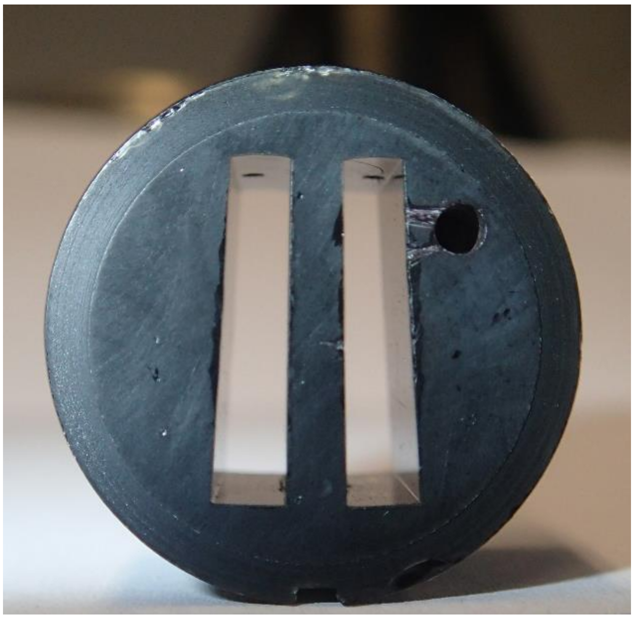

Figure S1. Photograph of a band-forming centerpiece with reference sector (left), sample sector (right) and reservoir.

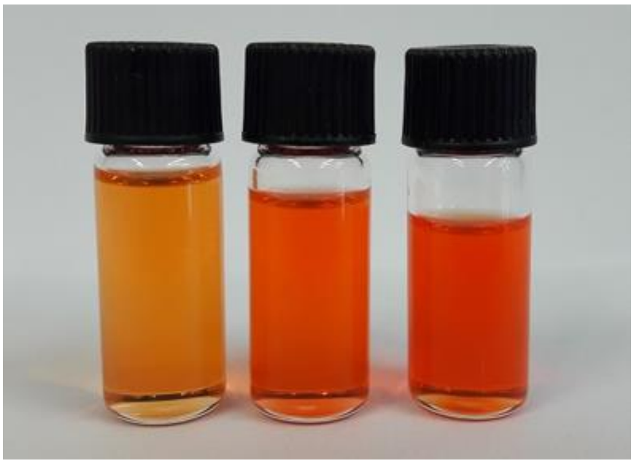

Figure S2. Photograph of CdSe nanoparticle dispersions. From left to right: small nanoparticles (d = 2.8 nm), medium-sized nanoparticles (d = 3.2 nm) and large nanoparticles (d = 3.8 nm).

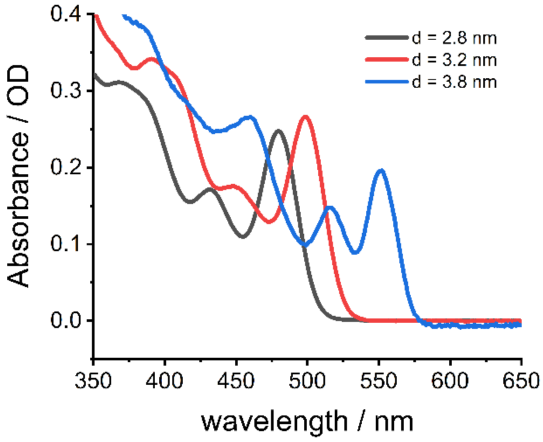

Figure S3. UV/Vis absorbance spectra of spherical CdSe nanoparticles with different diameters in toluene.

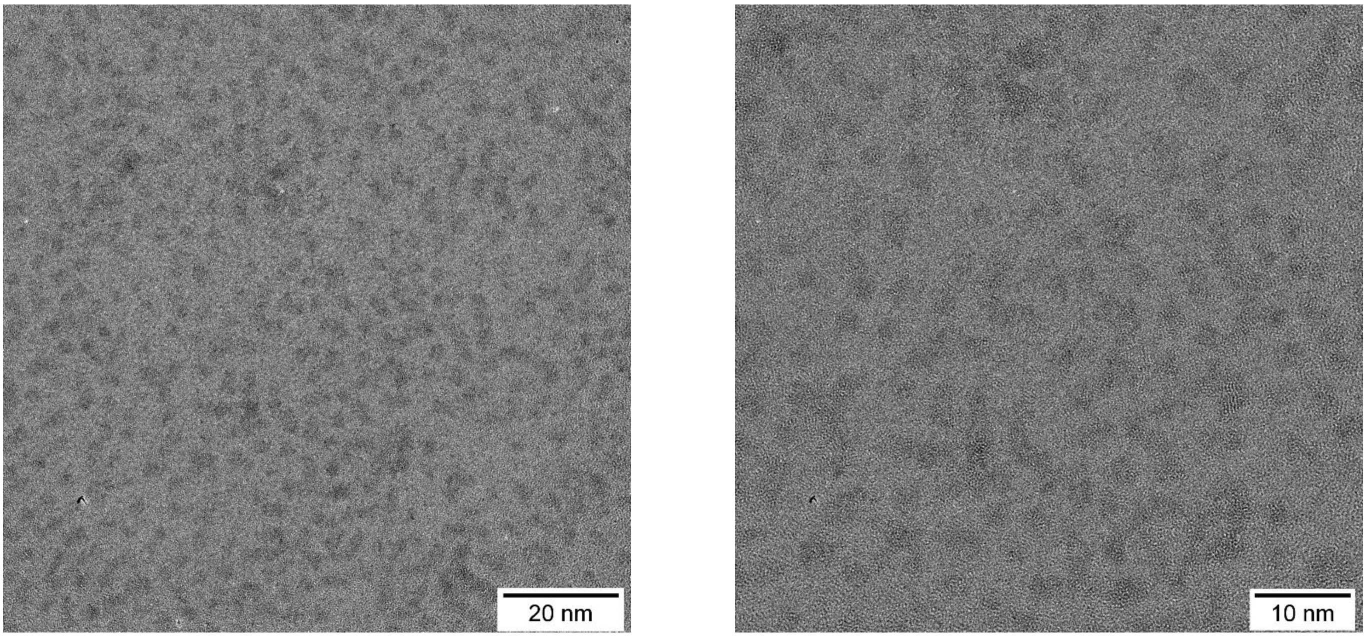

Figure S4. HR-TEM-Images of small spherical CdSe nanoparticles with a diameter of 2.8 nm.

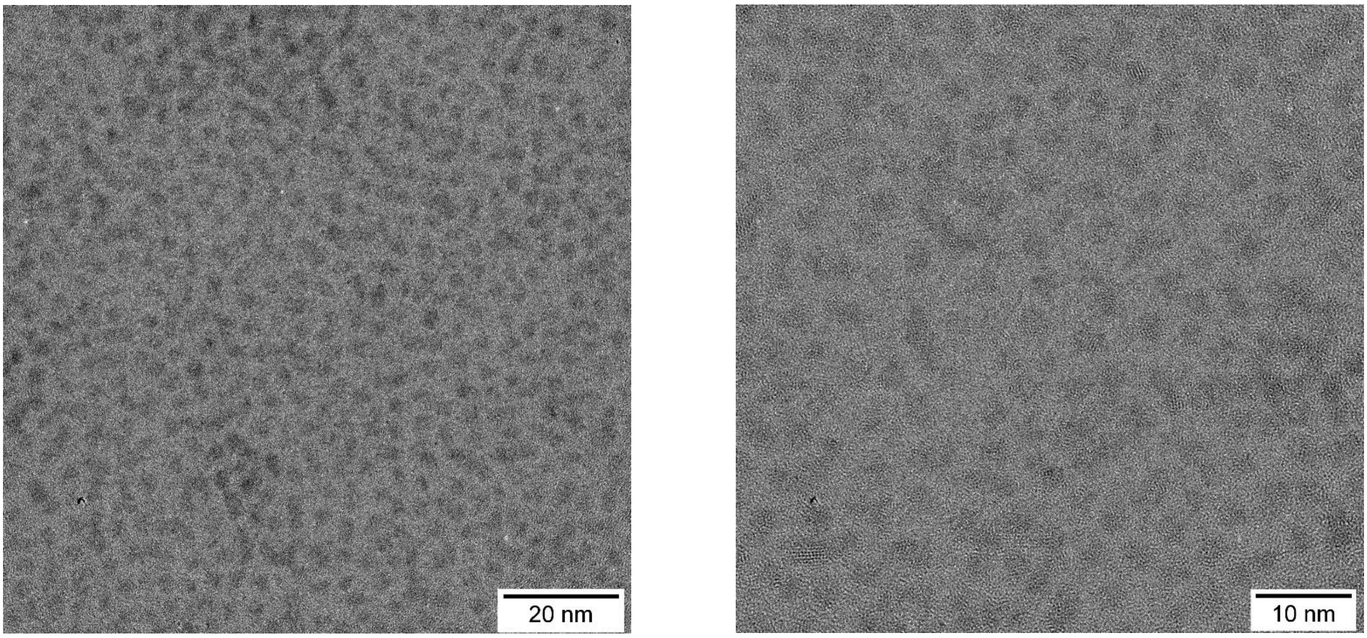

Figure S5. HR-TEM-Images of medium-sized spherical CdSe nanoparticles with a diameter of 3.2 nm.

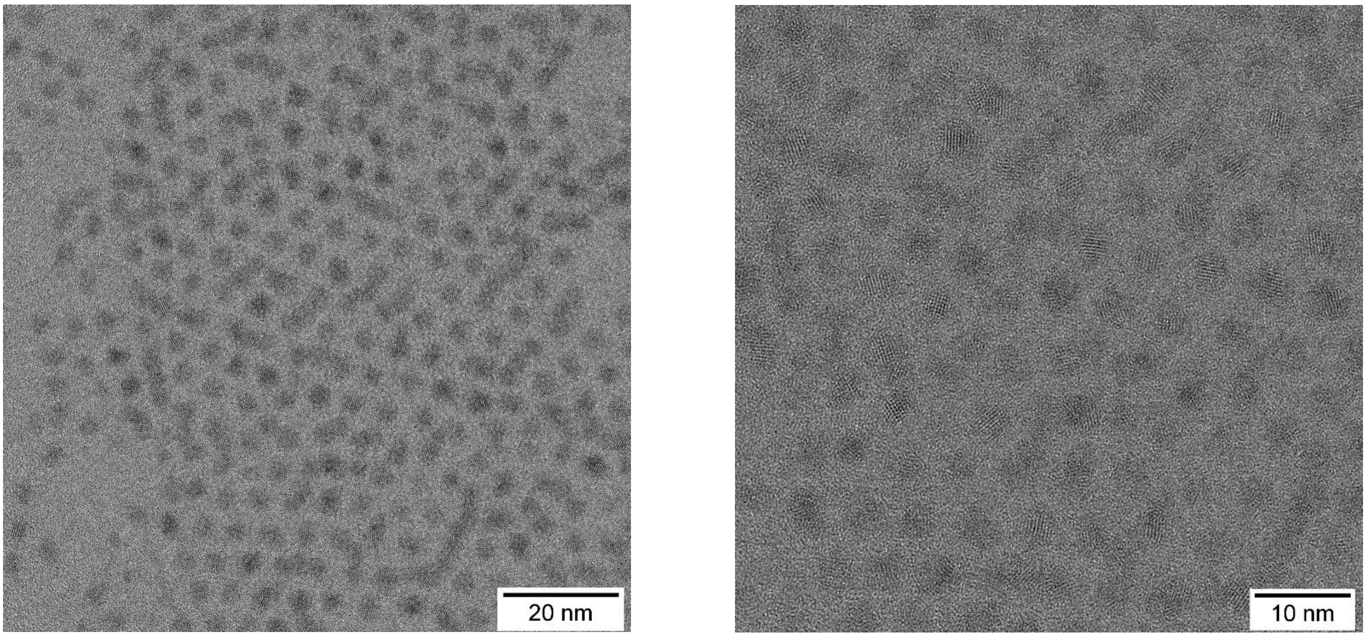

Figure S6. HR-TEM-Images of large spherical CdSe nanoparticles with a diameter of 3.8 nm.

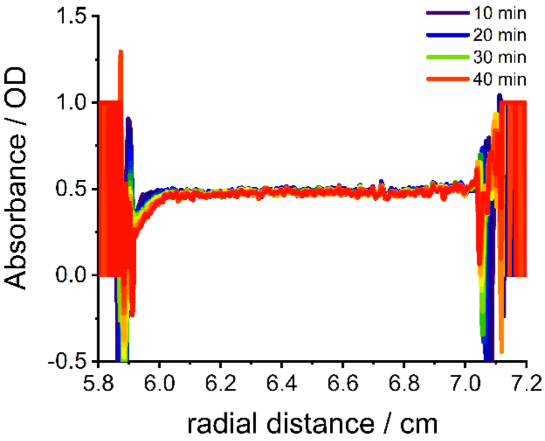

Figure S7. Absorbance profile of gelatin with large (d = 3.8 nm, λ = 546 nm) CdSe nanoparticles against radial distance from axis of rotation at different times in a sedimentation velocity experiment at 40 000 rpm (129 000 RCF(max)).

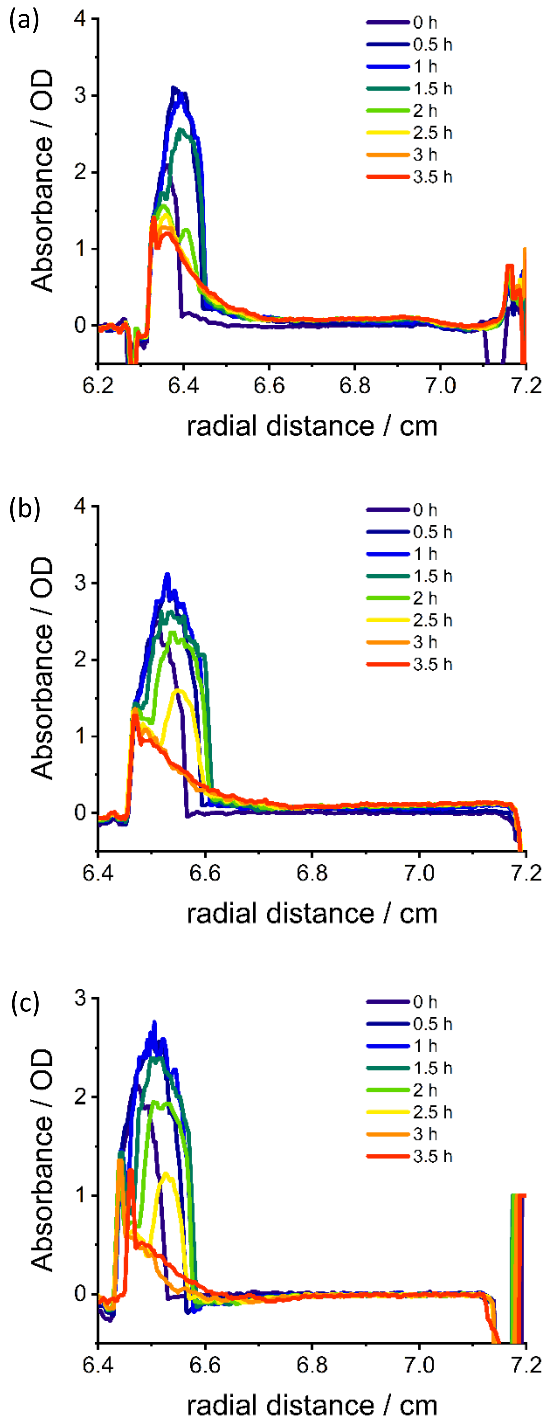

Figure S8. Absorbance profiles of gelatin with small (a) (d = 2.8 nm, λ = 482 nm), medium-sized (b) (d = 3.2 nm, λ = 500 nm) and large (c) (d = 3.8 nm, λ = 500 nm) CdSe nanoparticles against radial distance from axis of rotation at different times after overlaying at early times at 36 °C. Black band phenomena are observed.

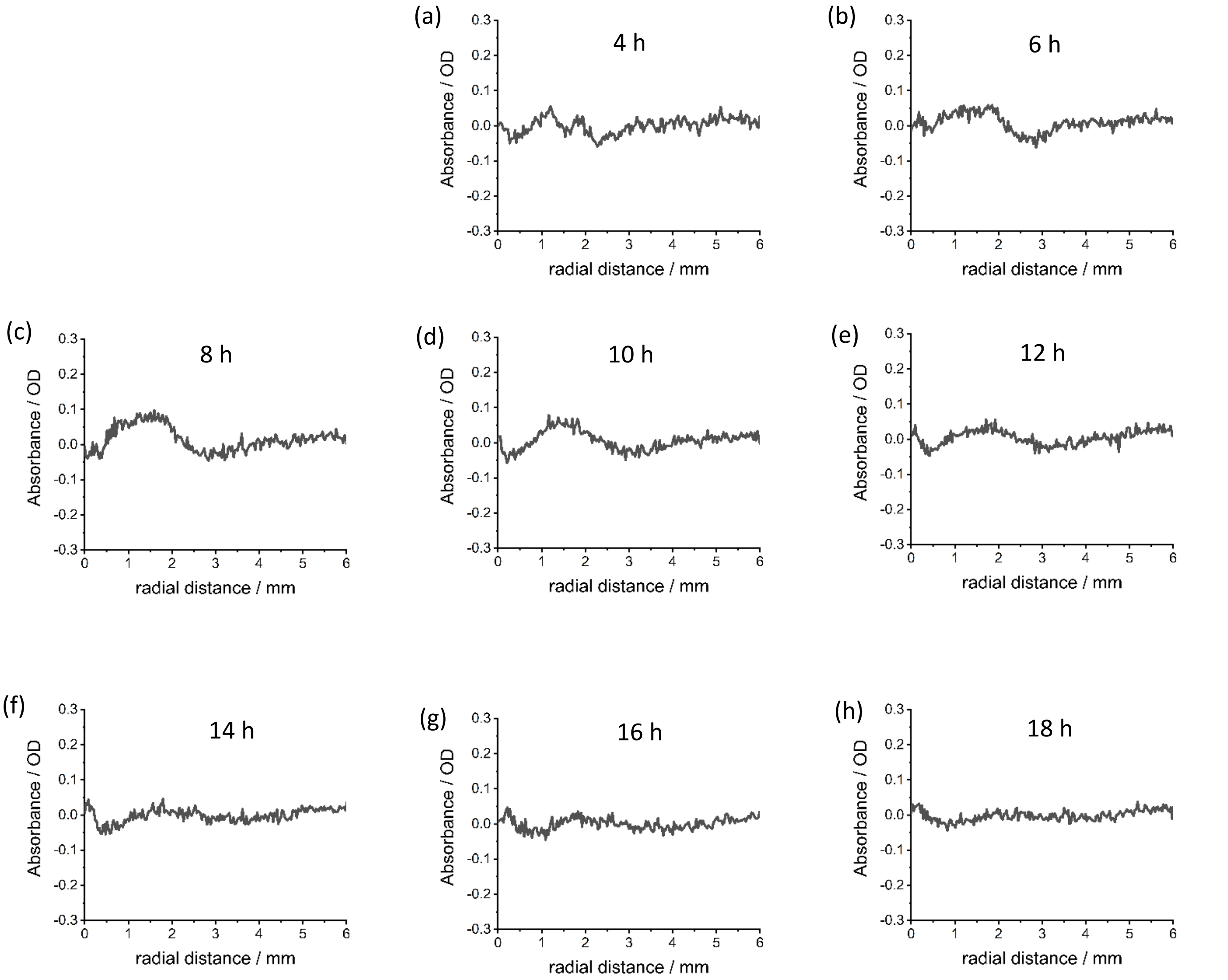

Figure S9. Absorbance residuals between simulation and experimental detection for gelatin with large (d = 3.8 nm) CdSe nanoparticles after (a) 4 h, (b) 6 h, (c) 8 h, (d) 10 h, (e) 12 h, (f) 14 h, (g) 16 h and (h) 18 h at λ = 500 nm at 36 °C. Radial distance is distance from the top of the polymer melt.

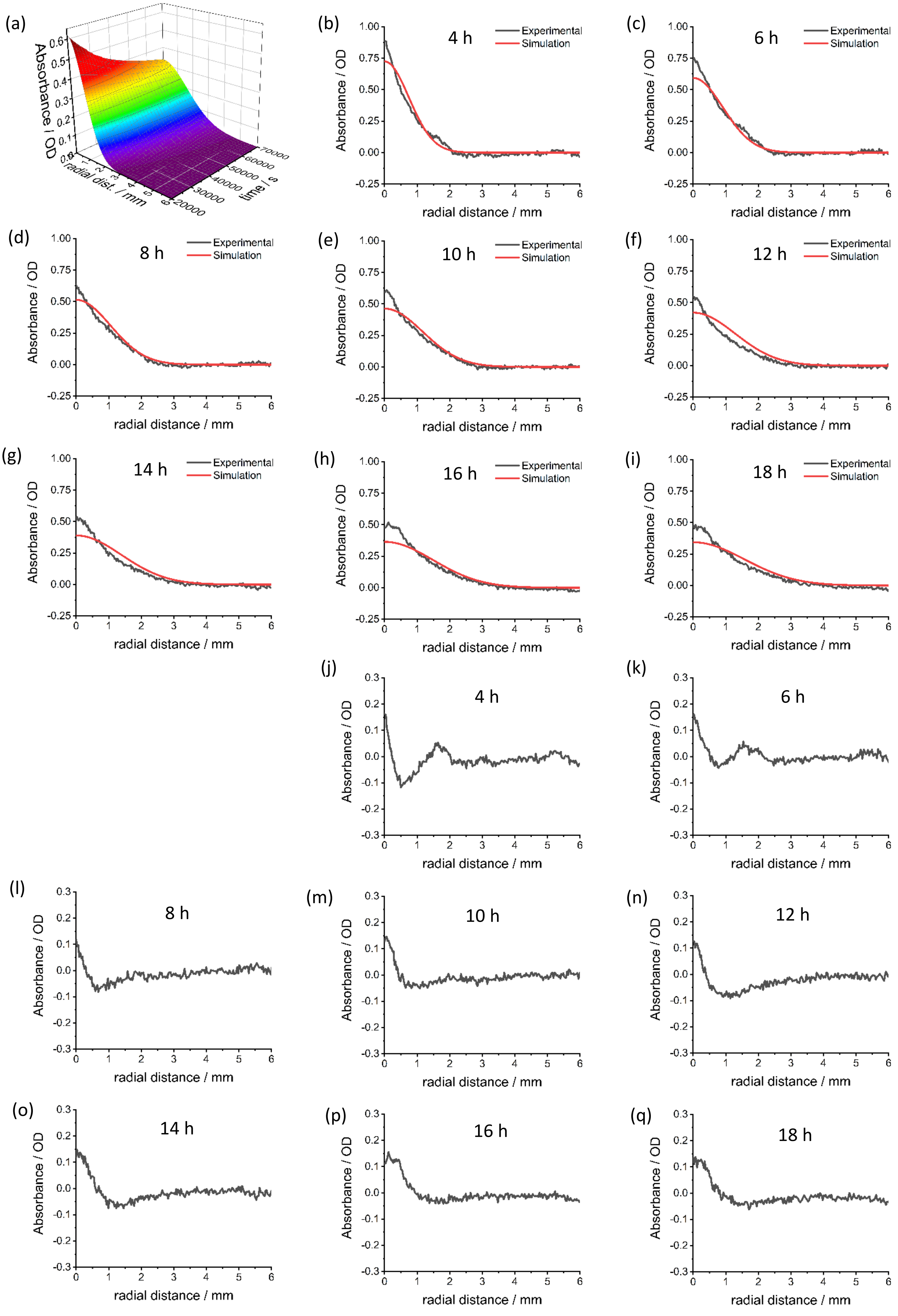

Figure S10. (a) Simulated absorbance profile after overlaying of small CdSe nanoparticles (d = 2.8 nm) over time; Comparison of simulated and experimental absorbance profiles after overlaying of small CdSe nanoparticles (d = 2.8 nm) after (b) 4 h, (c) 6 h, (d) 8 h, (e) 10 h, (f) 12 h, (g) 14 h, (h) 16 h and (i) 18 h at λ = 482 nm at 36 °C. Absorbance residuals between simulation and experimental detection for gelatin with small (d = 2.8 nm) CdSe nanoparticles after (j) 4 h, (k) 6 h, (l) 8 h, (m) 10 h, (n) 12 h, (o) 14 h, (p) 16 h and (q) 18 h at λ = 482 nm at 36 °C.

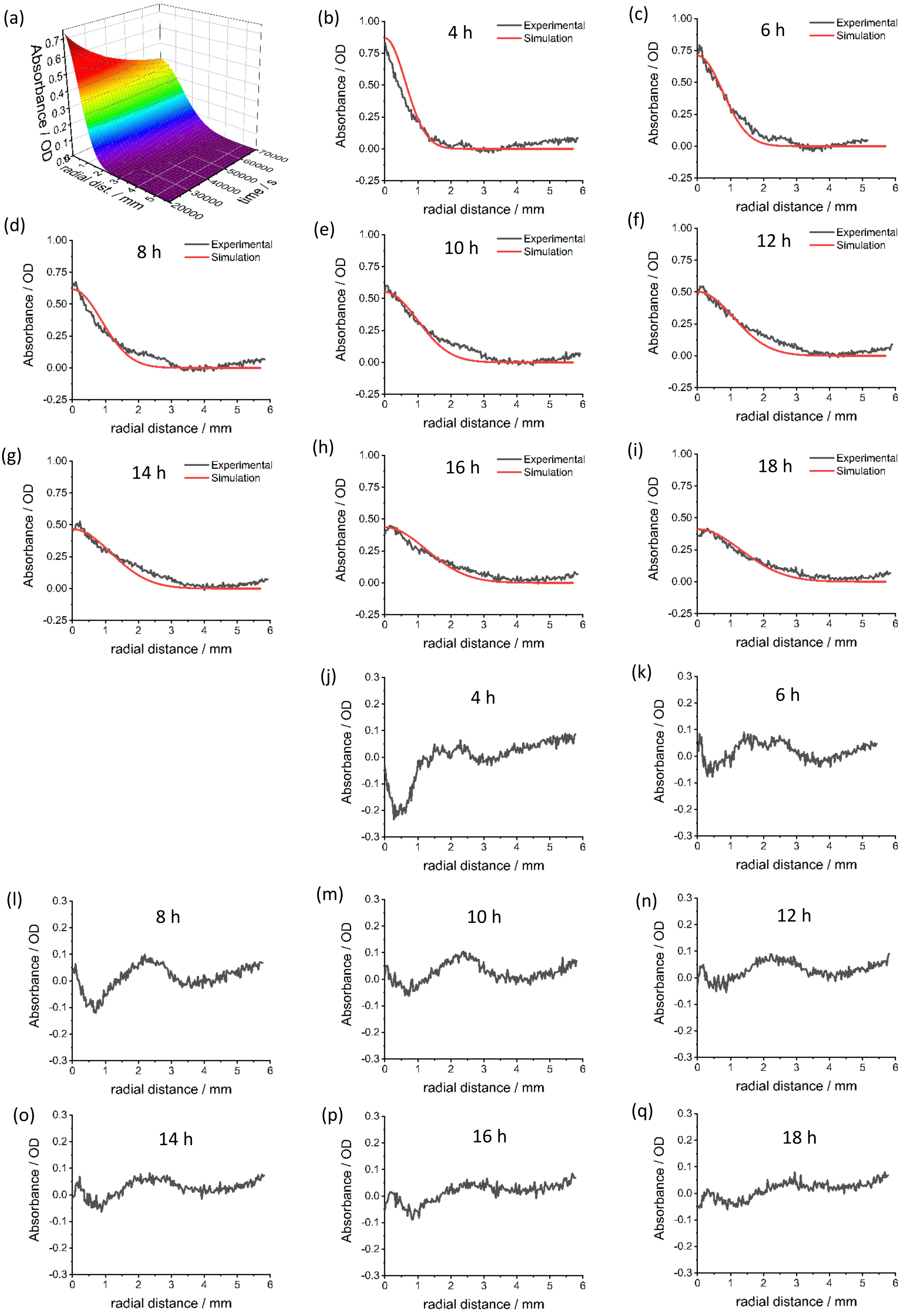

Figure S11. (a) Simulated absorbance profile after overlaying of medium-sized CdSe nanoparticles (d = 3.2 nm) over time; Comparison of simulated and experimental absorbance profiles after overlaying of medium-sized CdSe nanoparticles (d = 3.2 nm) after (b) 4 h, (c) 6 h, (d) 8 h, (e) 10 h, (f) 12 h, (g) 14 h, (h) 16 h and (i) 18 h at λ = 500 nm at 36 °C. Absorbance residuals between simulation and experimental detection for gelatin with small (d = 2.8 nm) CdSe nanoparticles after (j) 4 h, (k) 6 h, (l) 8 h, (m) 10 h, (n) 12 h, (o) 14 h, (p) 16 h and (q) 18 h at λ = 500 nm at 36 °C. Radial distance is distance from the top of the polymer melt.

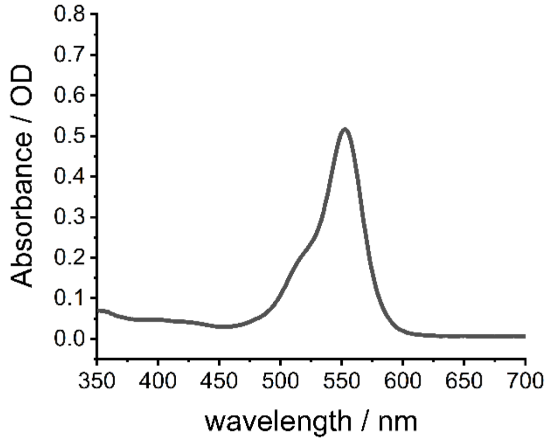

Figure S12. UV/Vis absorbance spectra of RITC-SiNPs (d = 25 nm) in water.

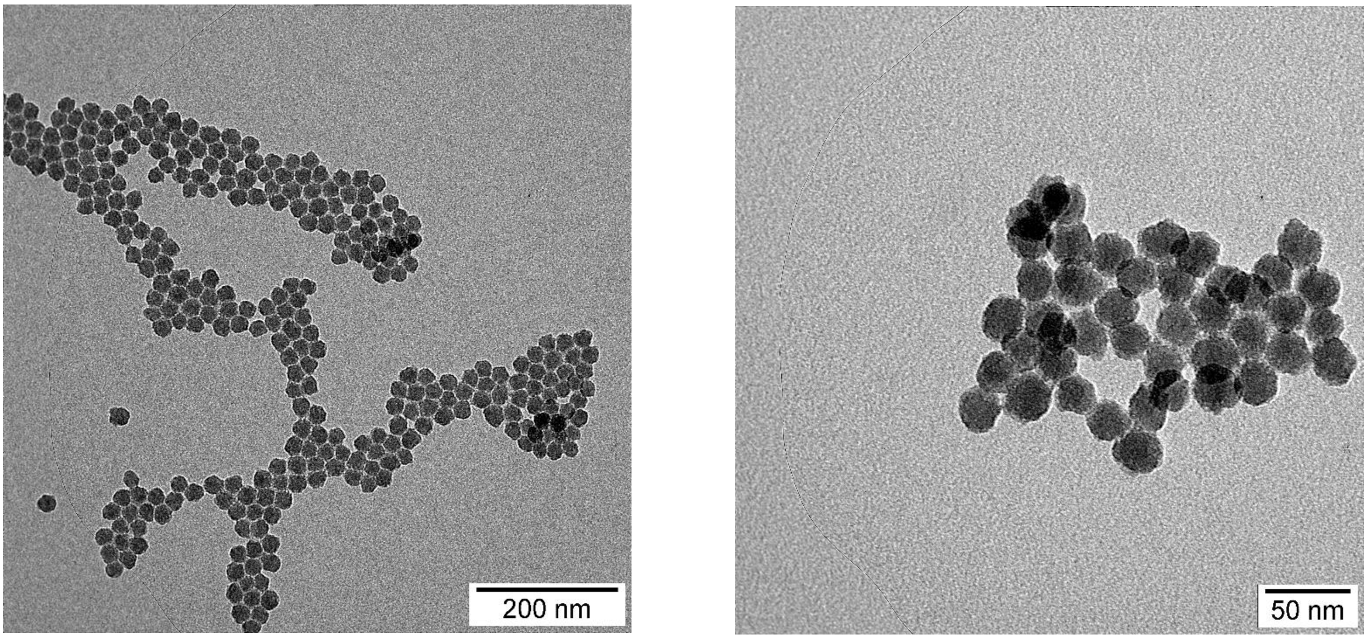

Figure S13. TEM-Images of spherical Rhodamine B isothiocyanate-incorporated silicananoparticles (RITC-SiNPs) with a diameter of 25 nm.

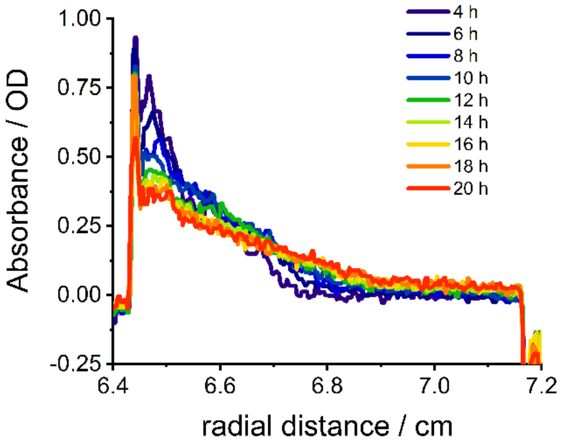

Figure S14. Absorbance profile of gelatin with RITC-SiNPs against radial distance from axis of rotation at different times after overlaying at λ = 550 nm at 40 °C.

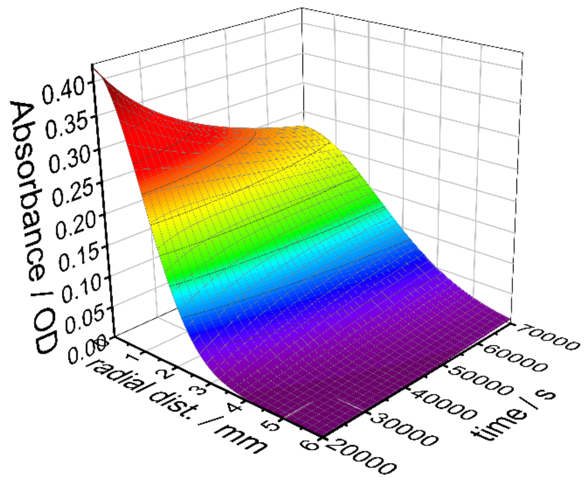

Figure S15. Simulated absorbance profile after overlaying of small RITC-SiNPs (d = 25 nm) over time. Radial distance is distance from the top of the polymer melt.

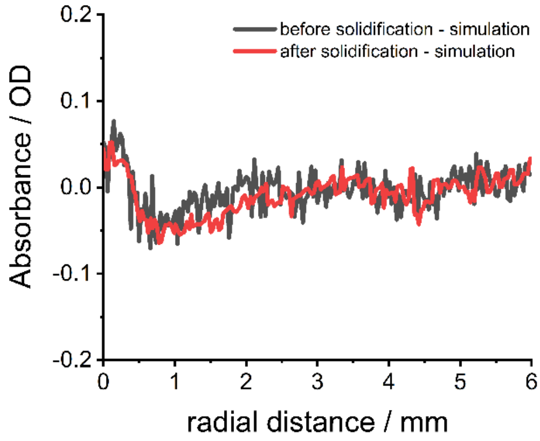

Figure S16. Absorbance residuals between simulation and experimental detection for nanoparticle gradient material with RITC-SiNPs (d = 25 nm) before and after solidification at λ = 550 nm. Radial distance is distance from the top of the polymer melt.

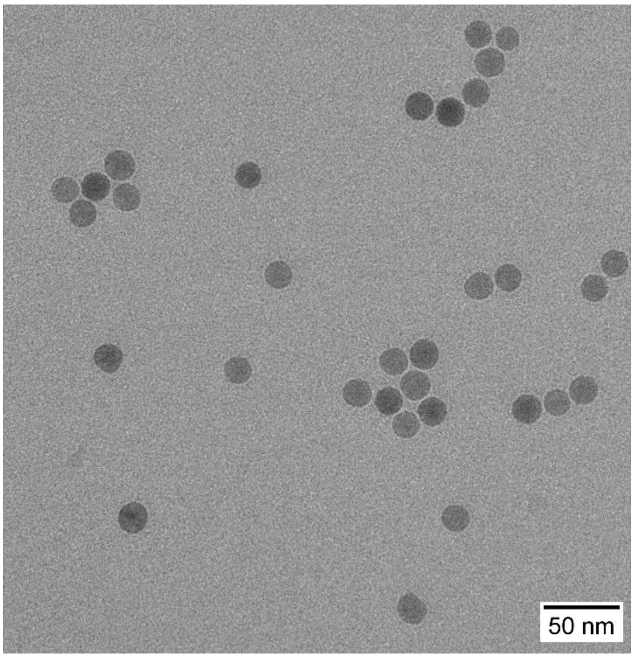

Figure S17. TEM-Images of spherical superparamagnetic iron oxide nanoparticles (SPIONs) with a diameter of 19 nm.
